# Machine learning framework for predicting the shear capacity of demountable bolted connectors in composite beams

**DOI:** 10.1038/s41598-025-28891-w

**Published:** 2025-12-04

**Authors:** Ahmed I. Saleh, Nabil S. Mahmoud, Fikry A. Salem, Mohammed Shaaban, Mohamed Ghannam

**Affiliations:** 1 Civil Engineering Department, Faculty of Engineering, Delta University for Science and Technology, Gamasa, Egypt; 2 Structural Engineering Department, Faculty of Engineering, Mansoura University, Mansoura, Egypt

**Keywords:** Demountable shear stud connectors, Ultimate shear capacity prediction, Linear regression, Ridge, Lasso, K-Nearest neighbors (KNN), Support vector regression (SVR), Decision tree, Random forest, XGBoost, Engineering, Materials science, Mathematics and computing

## Abstract

Steel–concrete composite beams are increasingly adopted in modern construction owing to their high strength, stiffness, and efficiency. Conventional welded shear connectors, while effective, hinder disassembly and recycling, limiting their alignment with sustainable construction practices. To address this, demountable bolted connectors have emerged as a viable alternative, promoting reuse, reduced waste, and compatibility with modular construction. This study presents eight machine learning algorithms including linear, tree-based, and ensemble methods were trained on a hybrid dataset combining experimental that were collected from previous studies and numerical results. Among these ML models, the XGBoost Regressor exhibited the highest accuracy (R² ≈ 0.996) with consistently low error margins, while SHAP-based interpretability confirmed bolt diameter and reinforcement strength as the most influential predictors. The findings highlight the potential of combining advanced testing, finite element modeling, and machine learning to establish robust predictive tools for demountable connector systems. This multidisciplinary approach not only improves design accuracy but also supports the development of sustainable, reusable, and high-performance composite structures in line with circular economy principles.

**Supplementary Information:**

The online version contains supplementary material available at 10.1038/s41598-025-28891-w.

## Introduction

Steel–concrete composite beams have become a fundamental element in modern structural systems because of their high strength, stiffness, and overall efficiency. Their performance depends largely on shear connectors, which transfer forces between steel and concrete so that the two materials act compositely. Conventional practice has relied on welded headed studs, but these permanent connectors hinder disassembly and material recovery, leading to waste generation and a loss of resource value at the end of a structure’s service life^1^. To address these issues, alternative demountable connectors have been introduced, typically using bolts, threaded fasteners, or friction-grip systems, thereby facilitating dismantling, recycling, and reuse^2–4^. Such advances are consistent with circular economy concepts, which promote design for disassembly, resource conservation, and recycling. Moreover, welded studs require energy-intensive installation, further elevating carbon emissions in the construction sector^5^.

Beyond sustainability, removable shear connectors provide economic advantages for modular and prefabricated building. Fast assembly and disassembly decrease time, labor, and waste^2^. Experimental studies have shown that bolted connectors can achieve comparable or superior ductility, load-slip response, and ultimate strength compared with welded studs, making them viable for high-demand applications including tall buildings and bridges^6^. For instance, Ataei et al.^7^ observed enhanced ductility and distinct failure mechanisms in bolted connectors applied to thin-walled beams, Fahmy et al.^8^ proved that bolt shape, grout strength, preloading, and material qualities all have a substantial impact on performance. Their work also demonstrated the possibility of reuse and reliability of such connectors, as well as enhanced prediction models. Several subsequent investigations have proposed new design approaches for demountable connectors. Ataei^9^ developed predictive equations that account for bolt clearance, preload, and material interaction, while Kwon et al.^10^ explored retrofit solutions using post-installed bolts. Pavlović et al.^11^ demonstrated that bolted connectors can achieve shear resistance comparable to welded studs, with the added advantage of easier dismantling.

Lam and Dai^12^ and Lee and Bradford^13^ investigated eco-friendly methods that combined geopolymer concrete with bolted connectors, resulting in enhanced ductility while lowering carbon emissions. Rehman, et al.^14^ Furthermore, these systems meet Eurocode 4’s ductility and strength standards^15^. Blind bolts and removable connections have lately been emphasized for their structural and environmental benefits in composite and precast applications. Similarly, Suwaed and Karavasilis^16^, Lam et al.^17^, and Patel et al.^18^ highlighted the structural and environmental benefits of blind bolts and disassembled connectors for composite and precast installations. On a similar note, Kozma et al.^19^, Loqman et al.^20^, and Hosseini et al.^21^ revealed that disassembled connectors In addition to allow reuse and deconstruction, but they also have higher fatigue strength and slip resistance than welded studs. Furthermore, Suwaed^22^ introduced a novel friction-based, high-strength bolted connector that combines high shear capacity with efficient disassembly. The development of these systems has been supported by advanced numerical modeling. Wang et al.^23^ Performed a finite element (FE) analysis to investigate bolted connectors under inverse push-off scenarios, while Csillag and Pavlović^24^ explored blind-bolted and SRR mechanisms for FRP decks. Bolted connectors in thin-walled beams have been validated by Hosseinpour et al.^25^ to meet Eurocode 4 ductility standards. Several new connector types have since arisen, including grout-filled lockbolt systems and conical lugs^26^, Y-shaped bolted connectors with tapered nuts, and double-nut friction-grip designs^27^. Song et al.^28^ proposed tapered iron bolts (TIBs) supported by new design models, whereas Liu et al.^29^ and Jakovljević et al.^30^ demonstrated the flexibility of embedded single-nut connectors in both precast and poured into place systems. Fang^31^ confirmed that high-strength bolts in UHPC exhibit better shear performance. In addition, studies by Fahmy et al.^8^ combined experimental and FEM analyses to provide predictive frameworks for load capacity and stiffness. Yu and Kim^32^ examined stud shear connectors in steel–UHPC systems, considering short stud geometry and nonlinear behavior such as head rotation and plastic deformation, leading to improved prediction accuracy.

Recent efforts also emphasize low-carbon design. Ataei et al.^33^ demonstrated that precast geopolymer slabs connected with friction-grip bolts achieve sufficient ductility and load capacity while enabling full deconstruction. Du et al.^34^ showed that prefabricated truss slabs with demountable bolts achieved comparable flexural performance to cast-in-place systems while improving efficiency and recyclability. Loqman et al.^35^ numerically validated that tongue-and-groove joints in precast slabs combined with larger bolts further enhance stiffness. Pardeshi and Patil^36^ reviewed connector systems comprehensively, concluding that demountable bolts provide a balance between structural performance and sustainability. Building on this, Luo and Yan^37^ proposed yielding bolts that preserved strength while enhancing ductility, and Fang et al.^38^ confirmed that bolted UHPC slab connections achieve greater ductility and disassembly potential than welded systems. Du et al.^39^ investigated high-strength steel–concrete beams, reporting that higher concrete grades enhance flexural performance, though conventional design methods tend to overestimate capacity.

Alongside these developments, machine learning (ML) has emerged as a valuable tool for predicting the shear behavior of connectors. However, limitations remain, including reliance on small or imbalanced datasets, neglect of cyclic or environmental effects, and a lack of transparency in model outputs. Many models provide predictions without interpretability, restricting their practical adoption in engineering. Moreover, few approaches integrate both experimental and numerical datasets to improve prediction accuracy.

Nevertheless, several successful ML applications have been reported. Zhu and Farouk^40^ applied neural networks optimized with particle swarm techniques to predict grouped stud connector resistance, surpassing conventional codes. Lee et al.^41^ found that artificial neural networks outperformed other ML models, identifying stud diameter, concrete strength, and yield strength as governing factors. Zhou et al.^42^ applied XGBoost and Random Forest to predict shear strength of studs in UHPC composites, using SHAP analysis to clarify variable importance. Li et al.^43^ developed an ensemble stacking model to predict the capacity of Perfobond Rib connectors, while Taffese et al.^44^ applied explainable AI methods to predict slip at steel–UHPC interfaces. For fatigue prediction, Kang et al.^45^ used Gaussian Process Regression to model connector life in bridges, offering more accurate results than existing design codes. He et al.^46^ reviewed ML applications for composite systems, noting both potential and limitations such as limited datasets and insufficient validation.

Although notable progress has been achieved, most existing models remain empirical and show limited applicability to the complex conditions associated with demountable systems. This underscores the need for advanced, data-driven frameworks that explicitly incorporate design variables, material characteristics, and performance criteria. Machine learning is an answer because it can effectively capture nonlinear and multivariate interactions that traditional methods frequently overlook. In this study, different machine learning models are constructed and evaluated using a hybrid dataset that includes both experimental findings and approved computational models of demountable bolted shear connectors.

The novelty of this work is to predict the allowable shear capacity for demountable shear stud connectors as most of previous work used the welded shear connectors, also an interactive software program was developed for calculating ultimate shear capacity, which will aid in the design of structurally efficient, sustainable, and reusable composites.

## Developed machine learning models

### Source of dataset

**Fig. 1:**
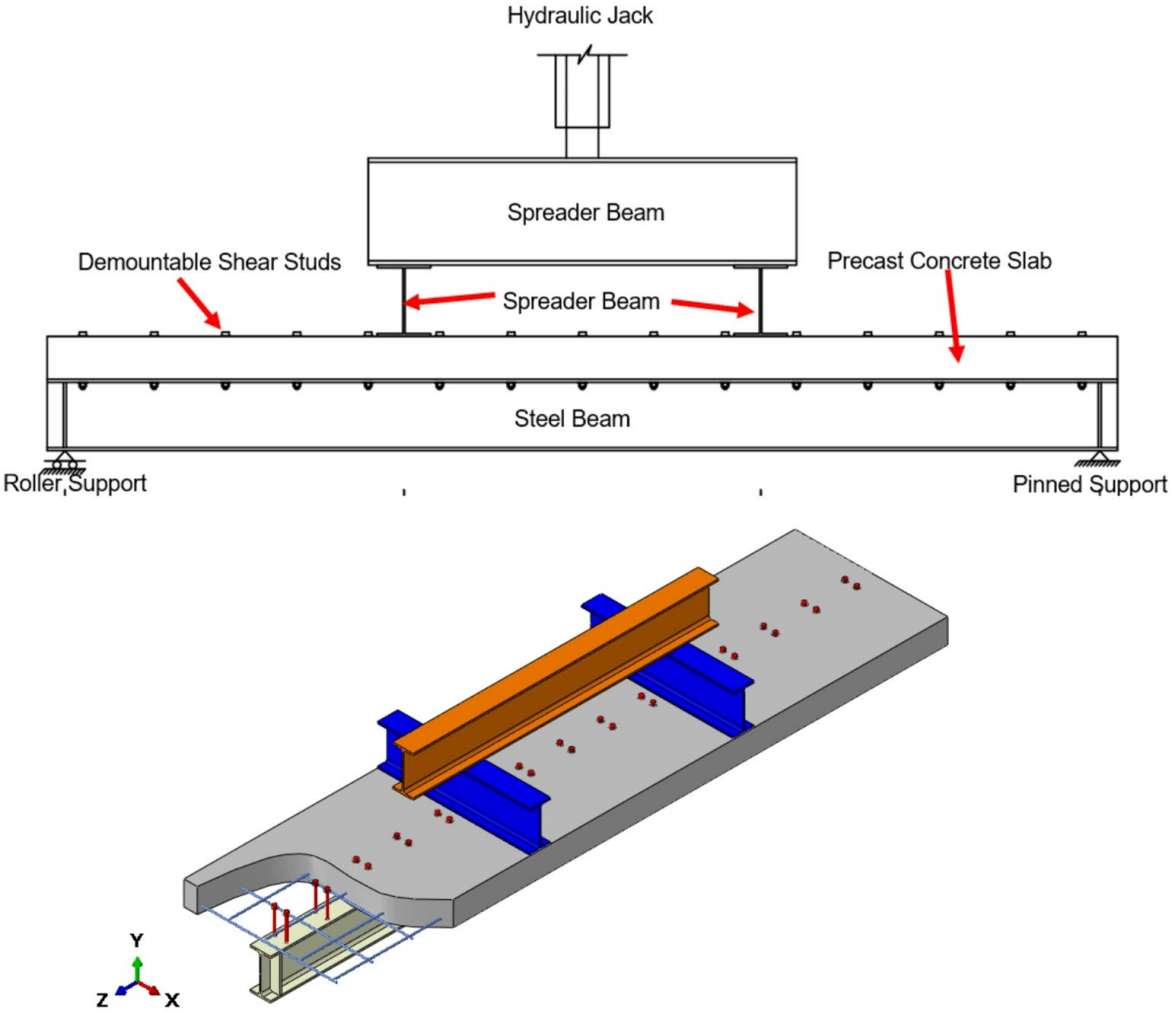
Schematic diagram of the test for demountable shear stud connectors.

The dataset utilized in this research integrates experimental findings from published studies with results obtained through validated finite element (FE) simulations. It covers a broad spectrum of demountable shear connector configurations, incorporating differences in geometry and material characteristics as shown in Fig. 1. The experimental component was derived from full-scale beam load–deflection tests, whereas the numerical data were produced using nonlinear FE models calibrated against experimental benchmarks to ensure accuracy. In total, 146 samples were compiled and provided as supplementary material, each representing a unique set of parameters affecting connector performance. Among these, 34 samples (23.29%) originated from experimental investigations, while 112 samples (76.71%) were generated numerically, as presented in Table 1.

Some data points in the main response variables deviate noticeably from the typical range, as detected using the interquartile range (IQR) approach. Specifically, fourteen observations showed unusual values for mid-span deflection, while sixteen did so for ultimate load capacity. These atypical measurements were considered valid reflections of the natural variability in the system rather than errors or anomalies. Although the dataset is dominated by finite element data, the experimental portion anchors the analysis in real-world structural behavior. To address the inherent scatter in experimental results, normalization techniques were applied. Multiple models were trained^47,48^ with cross-validation to reduce overfitting, each model is evaluated with shuffled K-fold cross-validation, using an adaptive 3–5 folds so splits stay reliable. Hyperparameter tuning runs on a separate 4-fold CV. To capture uncertainty, bootstrap: 1,000 resamples for overall metrics and feature-importance intervals. For reproducibility, random_state is set to 42 in the splitters and model initializations; to make the bootstrap pieces fully repeatable as well. Also, SHAP analyses and confidence intervals confirmed the robustness of the machine learning predictions. The issue of multicollinearity in our dataset was addressed by using Principal Component Analysis (PCA) to reduce feature redundancy through transformation into uncorrelated components. For modeling, ridge regression was applied to add stability by penalizing coefficient magnitude, helping control overfitting caused by correlated inputs. These methods were chosen for their suitability given the sample size and model type, balancing interpretability, and predictive performance effectively.

**Table 1:** Experimental vs. numerical data distribution.

Data type	Number of samples	Proportion, %
Experimental	34	23.29
Numerical	112	76.71
Total	146	100

### Selection of input parameters

A total of 13 input parameters were chosen to fully describe the geometric, material, and mechanical properties that affect the behavior of removable shear connectors in large-scale composite beams. The selection was informed by experimental evidence and engineering finding to ensure that the most influential features affecting load transfer and deformation capacity were captured. The geometric descriptors included the web thickness (t_w_), beam height (h), flange width (b_f_​), and flange thickness (t_f_), which together define the cross-sectional properties of the steel beam. The material-related features comprised the concrete compressive strength (f_cu, C​_), steel beam yield strength (f_y, S​_), reinforcement yield strength (f_yr_), and bolt yield strength (f_y, b​_), reflecting the stiffness and resistance of both the concrete matrix and steel components. Connector-related inputs were represented by the bolt diameter (D_b_​) and the number of bolts (n), while global structural parameters included the beam span (L) and stud spacing (pitch). To directly capture structural deformation, the midspan deflection was also included as an input parameter. The target output of the dataset was defined as the ultimate load capacity (P_u_​) of the connector system. By integrating geometry, material strengths, connection details, and deformation, this feature set provides a robust basis for developing machine learning models capable of accurately predicting the shear performance of demountable connectors in composite beams.

### Data normalization and splitting

Prior to training, the dataset was normalized to ensure that all input features contributed comparably to the learning process. The 13 input parameters varied in both units and magnitudes, for example, bolt diameter, compressive strength, and material strength making direct comparison problematic. To address this, each feature was rescaled to a range of 0 to 1 using the MinMaxScaler function from scikit-learn, where each value was divided by the corresponding feature’s maximum. After normalization, the 146 data samples were randomly shuffled to eliminate order-related bias and subsequently partitioned into two subsets: 70% for training and 30% for testing. This procedure ensured balanced model development and provided a reliable basis for evaluating predictive performance on unseen data.

### Evaluation metrics

For acquiring the models’ efficiency three regression metrics were applied: the coefficient of determination (R^2^) Eq. (1), Root Mean Square Error (RMSE) Eq. (2), and Mean Absolute Error (MAE) Eq. (3). Together, these indices offer a wide-ranging assessment of model accuracy, precision, and generalization. The R² value quantifies the proportion of variance in the dependent variable explained by the model, with values approaching unity indicating a strong agreement between predicted and observed responses. RMSE reflects the average magnitude of prediction errors, placing greater emphasis on larger deviations, which is particularly relevant in structural engineering applications where such discrepancies may be serious. By contrast, MAE calculates the mean absolute difference between predictions and observations, offering an easily interpretable measure of accuracy that is less sensitive to extreme values than RMSE.

1 $${R^2}=1 - \frac{{\sum {{\left( {{y_i} - {{\hat {y}}_i}} \right)}^2}}}{{\sum {{\left( {{y_i} - \bar {y}} \right)}^2}}}$$

2 $$MSE=\frac{1}{n}\mathop \sum \limits_{{i=1}}^{n} {\left( {{y_i} - {{\hat {y}}_i}} \right)^2}$$

3 $$MAE=\frac{1}{n}\mathop \sum \limits_{{i=1}}^{n} \left| {{y_i} - {{\hat {y}}_i}} \right|$$

where: ${y_i}$ are the detected values, ${\hat {y}_i}$ are the predicted values, $\bar {y}$ is the mean of the observed values.

### Statistical description

**Fig. 2:**
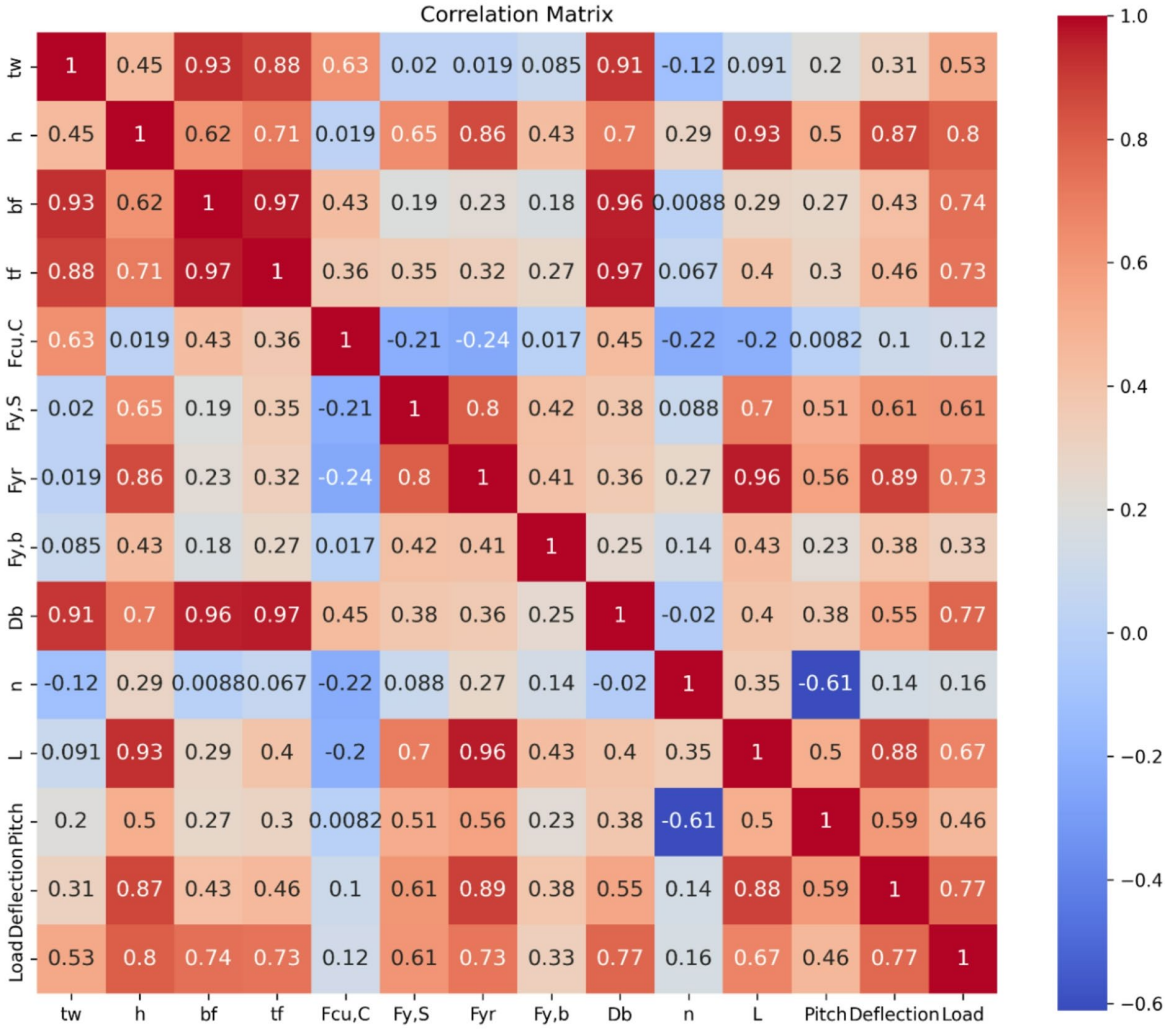
Pearson correlation among features.

Figure 2 reveals that several input variables significantly influence the load capacity. Among these, beam height (h), bolt diameter (D_b_), flange width (b_f_), and flange thickness (t_f_) all show strong positive correlations with load, having coefficients of 0.80, 0.77, 0.74, and 0.73, respectively. This highlights the critical role of increasing sectional dimensions in enhancing the beam’s ability to bear loads, consistent with fundamental structural principles. Material strength characteristics such as reinforcement yield strength (F_yr_) also exhibit a strong relationship with load (*r* = 0.73), indicating its importance in contributing to the structure’s strength and flexibility. On the other hand, concrete compressive strength (F_cu, C_) shows only a minimal association (*r* = 0.12), suggesting that its effect on load capacity is less pronounced when compared to steel reinforcement and connection features. The mid-span deflection correlates with load (*r* = 0.77), which aligns well with expected structural behavior under loading. Beam span (L) has a moderate correlation (*r* = 0.67), reflecting its impact on structural stiffness rather than direct load resistance. Additionally, there are strong interrelations among certain geometric properties, especially web thickness, flange width, and flange thickness, with correlations exceeding 0.90. This degree of multicollinearity points to the need for the application of techniques like dimensionality reduction or regularization in modeling to ensure more reliable and interpretable predictions.

Table 2 summarizes the descriptive statistics of the dataset and highlights the wide variability across the input features, which is essential for building robust (ML) models. For instance, the bolt diameter (D_b_) from 12 mm to 22 mm, while the number of bolts (n) ranges between 12 and 56, providing sufficient variation to capture how connector geometry influences load transfer. Similarly, the beam height (h), flange width (b_f_), and flange thickness (t_f_) cover a broad spectrum of values, reflecting different cross-sectional configurations that affect stiffness and strength.

Material properties also show considerable spread: the concrete compressive strength (f_cu, C_​) varies from 28.1 MPa to 145.45 MPa, while the steel yield strengths (f_y, S_ and f_yr_​) and the bolt yield strength (f_y, b_​) cover wide ranges, enabling the model to account for diverse material qualities. Global structural parameters, such as beam span (L) and stud spacing (Pitch), likewise demonstrate significant variation, ensuring that the dataset reflects different structural scales. The inclusion of midspan deflection, which ranges from 9.1 mm to 278 mm, further captures the deformation behavior under load.

The target variable, the ultimate load capacity (P_u_​), ranges from approximately 246 kN to 1246 kN, indicating that both relatively weak and strong connection performances are represented.

**Table 2:** Descriptive statistics of the numerical features.

Parameter	Mean	STD	Min.	25%	50%	75%	Max.
Input							
* t*_*w*_, *mm*	6.86	2.43	5.6	5.6	5.6	8	14
* h*,* mm*	234.86	69.47	200	200	200	250	454
* b*_*f*_, *mm*	133.05	54.42	100	100	100	190	255
* t*_*f*_, *mm*	9.84	2.14	8.5	8.5	8.5	12	14
* F*_*cu, C*_, *MPa*	47.32	27.38	28.1	28.65	40	60	145.45
* F*_*y, S*_, *Mpa*	287.44	33.21	256	280.3	280.3	280.3	404
* F*_*yr*_, *Mpa*	350.94	56.7	289.77	335.9	335.9	335.9	543
* F*_*y, b*_, *MPa*	646.32	208.61	308.6	464	640	900	936
* D*_*b*_, *mm*	14.13	3.61	12	12	12	16	22
* n*	23.88	8.89	12	15	20	30	56
* L*,* mm*	3351.94	1083.46	2400	3000	3000	3000	7000
* Pitch*,* mm*	309.94	129.36	200	200	300	400	777.78
* Deflection*,* mm*	63.95	52.92	9.12	42	48	60	278
Output							
* P*_*u*_ *(kN)*	398.92	224.25	246.41	279.76	294.91	450.4	1246.2

### Prediction performance of machine learning models

**Fig. 3:**
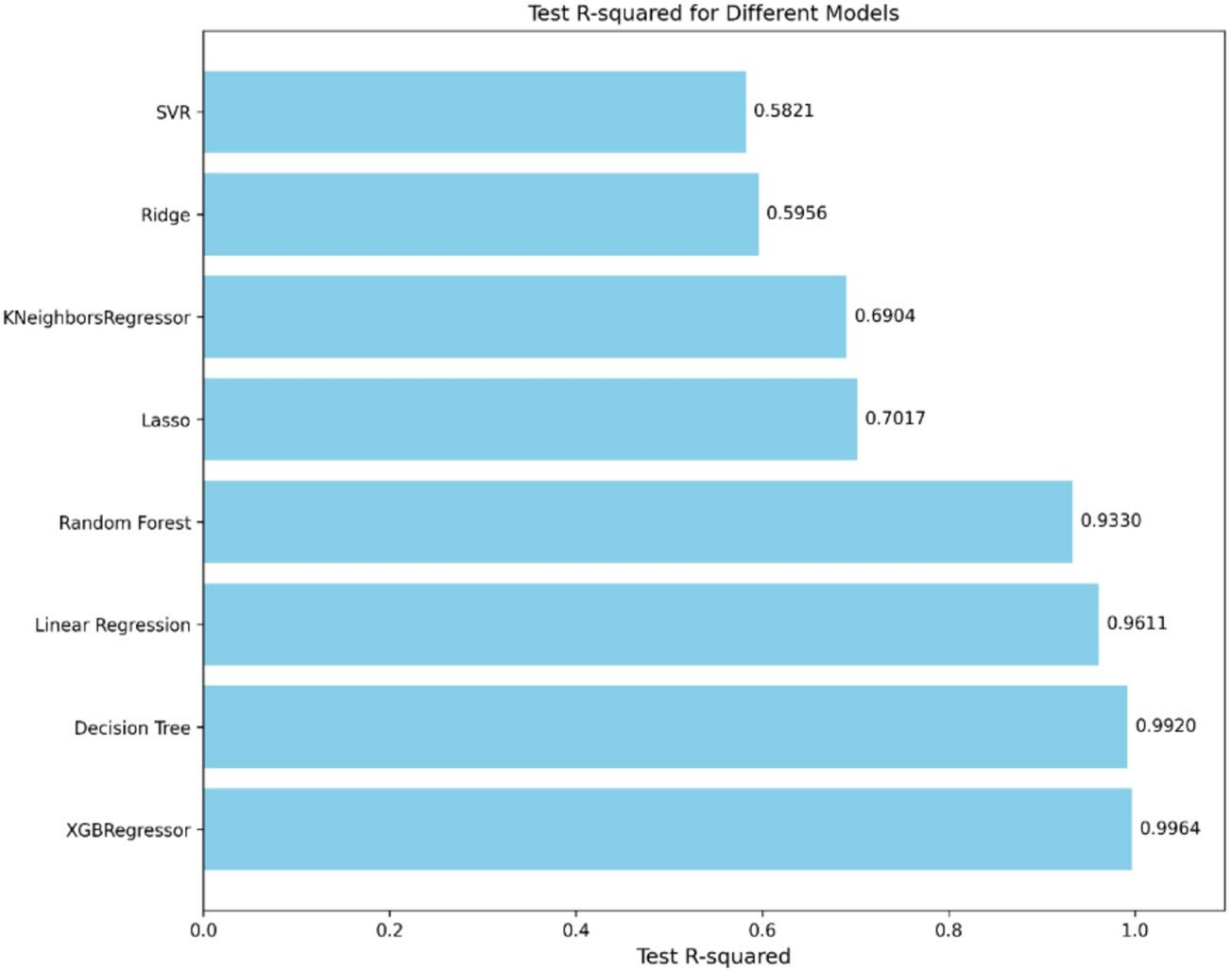
Tested R2 for different models.

The ML models assessed in the study vary in complexity and suitability for engineering applications. Linear models like Linear Regression, Ridge, and Lasso provide fast training and easy interpretation but may not capture complex, nonlinear behaviors. Tree-based methods such as Decision Trees and Random Forests handle nonlinearity better and often improve accuracy, though they demand more computation and are less transparent. K-Nearest Neighbors and Support Vector Regression adapt to complex patterns but can be computationally intensive and sensitive to tuning, limiting their practicality in some scenarios. XGBoost offers the best predictive performance but requires longer training times and more computing power, making it less suitable where quick updates or limited resources are factors. Selecting the appropriate model depends on balancing accuracy, interpretability, computational resources, and dataset size to best fit engineering needs. The results of the model comparison are summarized in Fig. 3, which reports the coefficient of determination (R^2^) for each regression technique in predicting the ultimate load capacity of demountable shear connectors. The XGBoost Regressor achieved the highest accuracy with an R^2^ of 0.9964, explaining all of the variance in the dataset. The Decision Tree Regressor also performed exceptionally well (R^2^ = 0.9920), highlighting the effectiveness of tree-based approaches in capturing nonlinear patterns and feature interactions. Linear Regression demonstrated a robust performance as well (R^2^ = 0.9611), showing that the data still retained an appreciable degree of linearity.

**Fig. 4:**
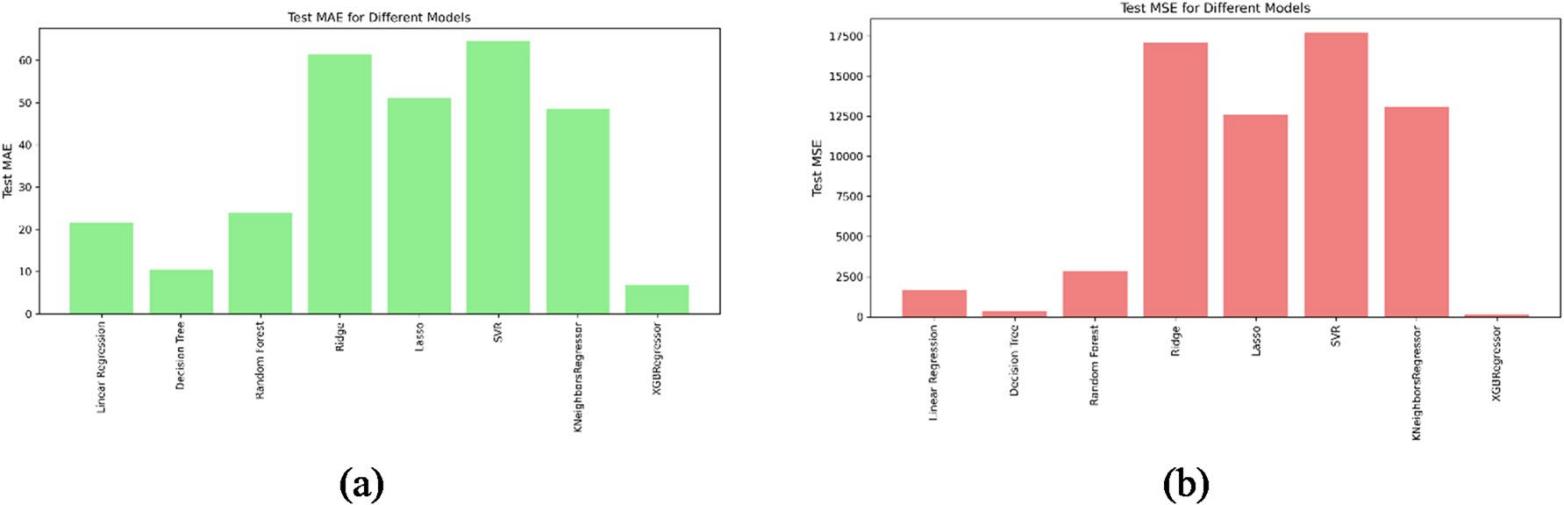
Bar charts for (a) Mean absolute error. (b) Mean squared error.

Figure 4 (a) presents the Mean Absolute Error (MAE) values for the regression models. MAE provides an intuitive measure of accuracy by showing the average deviation between predicted and actual load capacities. The XGBoost Regressor achieved the best performance, with the lowest MAE (≈ 7), indicating consistently precise predictions. The Decision Tree model also performed strongly, with a slightly higher but still low error level. Random Forest followed with moderate MAE values, confirming its reliability in reducing average prediction error. In contrast, Ridge and SVR reported the highest MAE values (above 60), showing weaker performance. Lasso and K-Nearest Neighbors (KNN) fell in the middle range, performing better than Ridge and SVR but less accurately than tree-based models.

Figure 4 (b) illustrates the Mean Squared Error (MSE) results, which penalize larger deviations more heavily, making them useful for assessing robustness. Once again, the XGBoost Regressor achieved the lowest MSE (close to zero), confirming its superior stability and resilience to outliers. The Decision Tree model produced comparably low MSE values, while Random Forest showed moderate error levels. On the other hand, SVR and Ridge Regression recorded the highest MSE values (exceeding 15,000), highlighting their reduced ability to generalize effectively. Lasso, Linear Regression, and KNN produced intermediate MSE values, reflecting moderate predictive capability.

Table 3 summarizes the 95% confidence intervals (CI) for R^2^, Mean Squared Error (MSE), and Mean Absolute Error (MAE) across the evaluated regression models. The results confirm that the XGBoost Regressor achieved the most reliable performance, with an R^2^ CI of 0.9833–0.9989, coupled with the lowest error ranges (MSE CI: 60.85–273.25, MAE CI: 4.09–10.03). The Decision Tree model followed closely, showing a strong R^2^ CI of 0.9626–0.9972 and low error bounds (MSE CI: 158.44–524.27, MAE CI: 6.14–15.07).

By comparison, the Linear Regression model demonstrated moderate reliability (R^2^ CI: 0.7902–0.9949), with relatively low error ranges, making it competitive but less consistent than tree-based approaches. The Random Forest model showed wider uncertainty (R^2^ CI: 0.6056–0.9854), with higher error variability, reflecting some instability in predictions.

The weaker-performing models were Ridge, Lasso, SVR, and K-Nearest Neighbors, all of which exhibited broad confidence intervals and lower R^2^ estimates. For example, Ridge regression had an R^2^ CI extending into negative values (–1.1047 to 0.8963) with high error bounds (MSE CI: 4929.05–31985.33), indicating poor fit and high variability. Similar trends were observed for Lasso, SVR, and KNN, all of which displayed inconsistent predictive power.

Overall, the confidence interval analysis reinforces that XGBoost and Decision Tree are the most effective and reliable models, providing both high accuracy and low variability, while linear regularized models and kernel-based methods demonstrated weaker and less stable performance.

**Table 3:** Confidence intervals for all models.

Model name	*R*^2^ CI (95%)	MSE CI (95%)	MAE CI (95%)
Linear regression	0.7902–0.9949	250.76–3674.87	12.78–33.18
Decision tree	0.9626–0.9972	158.44–524.27	6.14–15.07
Random Forest	0.6056–0.9854	793.21–5514.05	11.77–39.15
Ridge	-1.1047–0.8963	4929.05–31985.33	30.39–97.33
Lasso	-0.4756-0.9351	3270.46–24830.09	23.69–84.90
SVR	-0.1652-0.8488	4260.81–34801.83	34.84–100.44
KNeighborsRegressor	-0.0448-0.8993	2350.65–25810.46	20.36–81.21
XGBRegressor	0.9833–0.9989	60.85–273.25	4.09–10.03

### Machine learning models versus experimental results

**Fig. 5:**
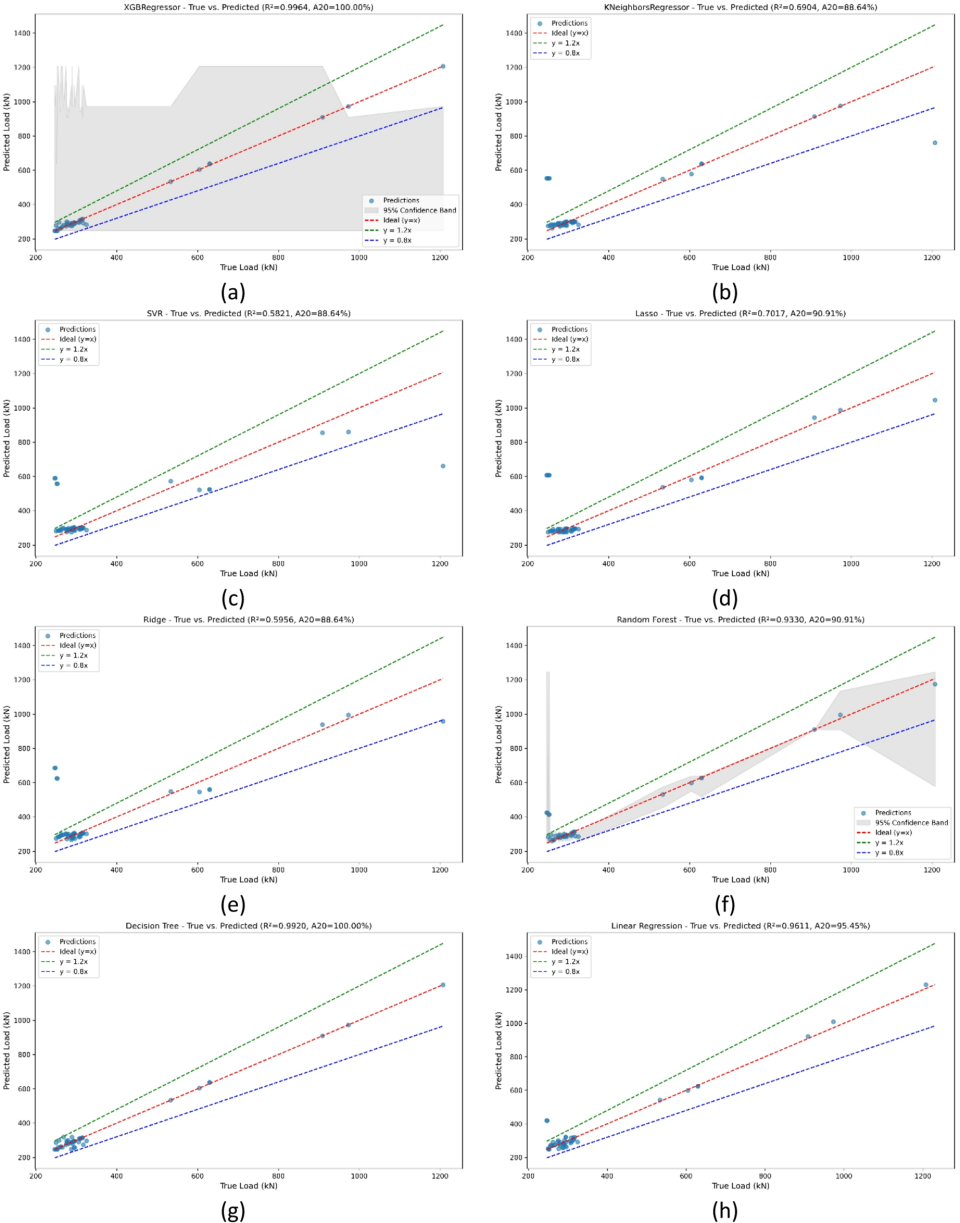
Predicted shear resistance from ML models versus experimental results. (a) XGBRegressor – True vs. Predicted, (Pu), (b) KNeighborsRegressor -True vs. Predicted, (Pu), (c) SVR – True vs. Predicted, (Pu), (d) Lasso - True vs. Predicted, (Pu), (e) Ridge - True vs. Predicted, (Pu), (f) Random Forest - True vs. Predicted, (Pu), (g) Decision Tree - True vs. Predicted, (Pu), (h) Linear regression - True vs. Predicted, (Pu)

Figure 5 presents the scatter plots of predicted v actual ultimate load (P_u_​) for the 8 regression models, allowing direct comparison of their predictive accuracy. The red dashed line represents the ideal fit (y = x), while the green and blue lines (y = 1.2, y = 0.8x) define acceptable tolerance bands.

The XGBoost Regressor (Fig. 5a) gives an R^2^ of 0.9964 and an A20 of 100%, showing that all predictions fell within ± 20% of the true values. The Decision Tree model (Fig. 5f) also performed remarkably well (R^2^ = 0.9920, A20 = 100), with predictions tightly clustered around the ideal line. The Linear Regression model (Fig. 5h) produced a strong fit (R^2^ = 0.9611, A20 = 95.45), demonstrating that even a simple linear model was able to capture much of the dataset’s structure.

The Random Forest (Fig. 5b) followed with an R^2^ of 0.9330 and A20 = 90.91, though its prediction spread was wider than that of XGBoost and Decision Tree. Regularized linear models such as Lasso (Fig. 5g), R^2^ = 0.7017 and Ridge (Fig. 5c) R^2^ = 0.5956 achieved only moderate accuracy, capturing the general trend but with significant deviations at higher loads. The K-Nearest Neighbors Regressor (Fig. 5e) performed similarly (R^2^ = 0.6904), with noticeable scatter around the diagonal.

The weakest predictive capability was observed for Support Vector Regression (Fig. 5d), with an R^2^ of 0.5821 and A20 = 88.64. The wide scatter and deviation from the ideal fit highlights its restrained ability to generalize to this structural dataset.

**Fig. 6:**
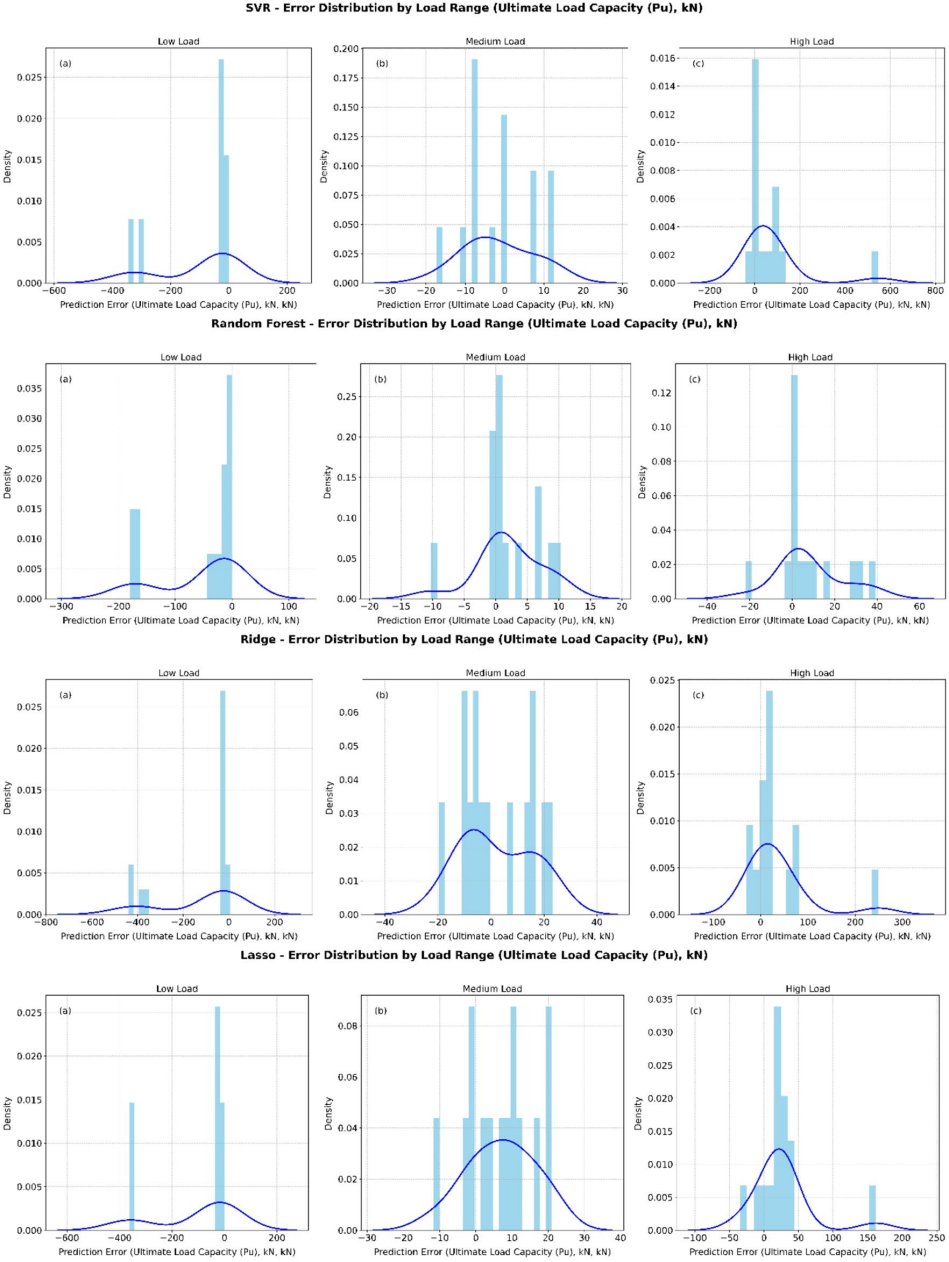

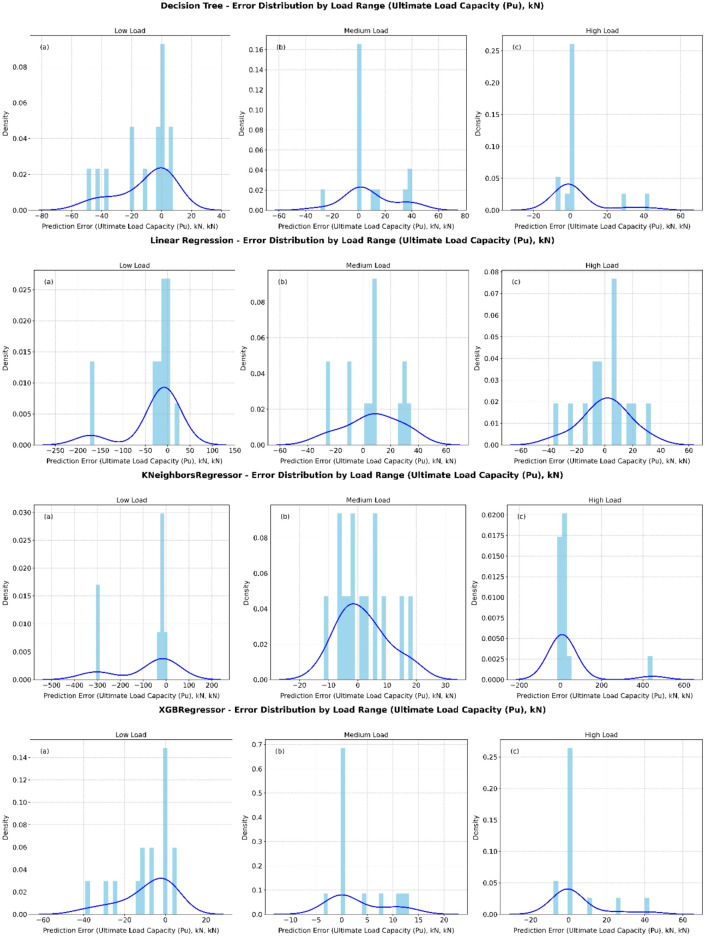
Error histograms for all models across different load ranges.

The error distribution plots in Fig. 6 compare Decision Tree, XGBoost Regressor, KNeighborsRegressor, Lasso, Linear Regression, Random Forest, Ridge, and SVR across low, medium, and high load levels. At low load, all models show a sharp concentration of errors near 0 kN, but with long negative tails (e.g., Decision Tree and XGBoost reaching about 100 kN), suggesting a tendency to underestimate load despite high accuracy around zero. Under medium load conditions, most models (such as XGBoost, Random Forest, Ridge, and SVR) present nearly symmetric, bell-shaped error distributions centered close to 0 kN, indicating more balanced predictions. However, some methods (e.g., Lasso and Linear Regression) still produce larger negative deviations, extending below 100 kN. For high load ranges, the distributions shift towards positive errors, reflecting a bias toward overestimation. Decision Tree and Random Forest show errors concentrated between 0 and 60 kN, while SVR and XGBoost produce the narrowest and most concentrated peaks, pointing to superior precision in this regime.

### SHAP-based explanation of ML models

**Fig. 7:**
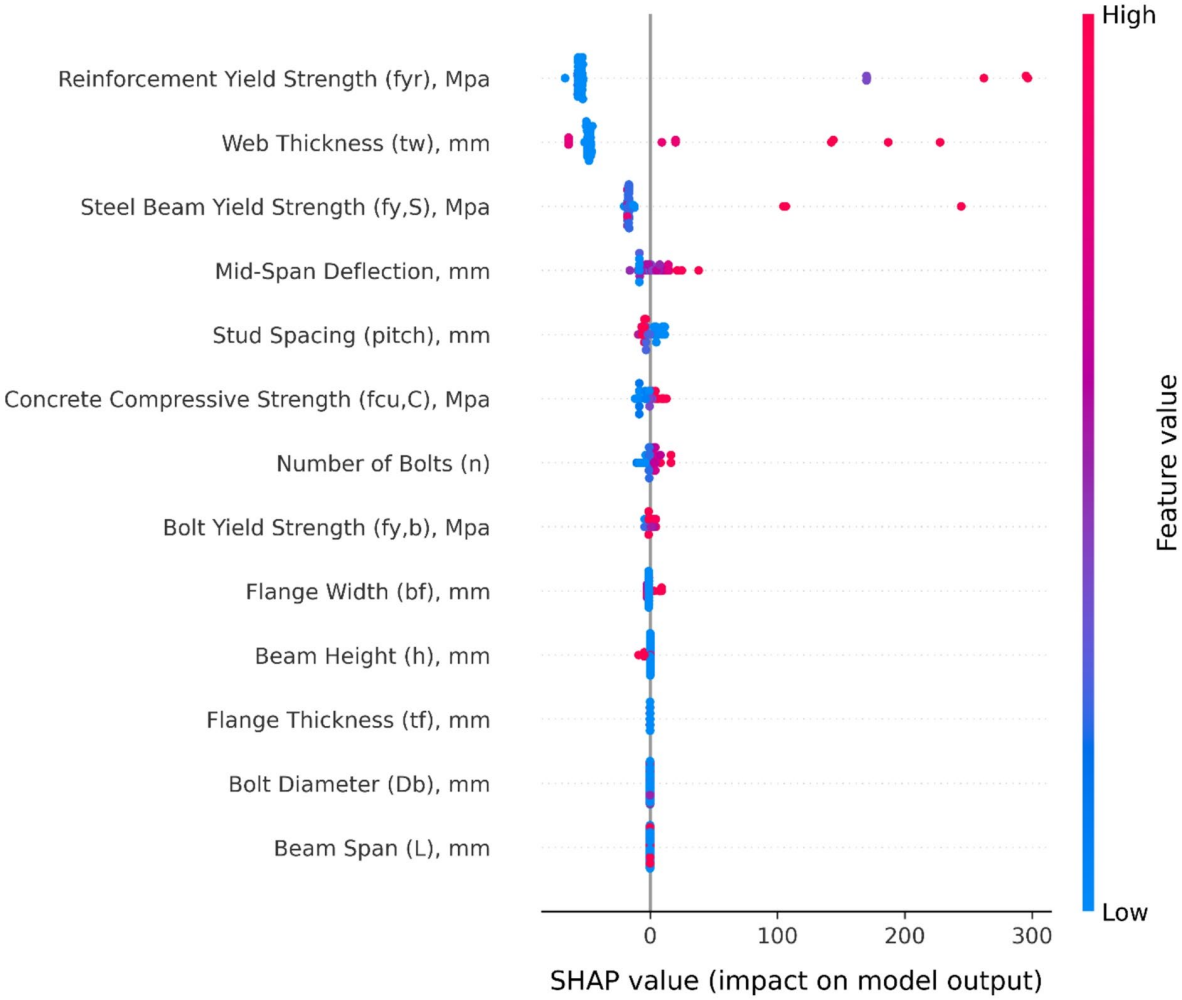
SHAP summary plot.

Figure 7 depicts a SHAP summary figure, which clearly shows how each input parameter affects the anticipated ultimate shear load (P_u_). In this representation, each dot signifies a solo forecast, with its horizontal placement representing the feature’s contribution (positive or negative) to the model output, and the color scale reflecting the feature magnitude from low (blue) to high (red).

The results highlight that bolt diameter (D_b_​) exerts the strongest influence on model predictions, with larger diameters consistently increasing the predicted load. This aligns well with engineering principles, as greater bolt size enhances the shear-resisting capacity. Other parameters with notable contributions include reinforcement yield strength (F_yr_), web thickness (t_w_), and steel yield strength (F_y, S_​), which demonstrate clear upward shifts in predicted load at higher values.

Geometric features such as deflection, pitch, and flange width (b_f_) also show moderate but consistent effects, indicating that spacing and deformation-related characteristics play a role in load transfer. Meanwhile, material strength parameters such as concrete compressive strength (F_cu, C_) and bolt yield strength (F_y, b_​) contribute positively, though with more scattered impacts compared to D_b_.

The low SHAP contribution of (F_cu, C_) does not imply that concrete is unimportant; rather, once composite action is established, concrete in compression is rarely the limiting component for the ranges observed. Connector detailing and slab geometry cap how much of the concrete capacity can be mobilized; beyond that threshold, higher (F_cu, C_) yields diminishing returns, which the model registers as a small marginal impact. A similar argument holds for (t_f_): its influence on section modulus is often overshadowed by member depth and flange width, and by stability effects that are more sensitive to web slenderness than to incremental flange thickening.

### Feature importance

**Fig. 8:**
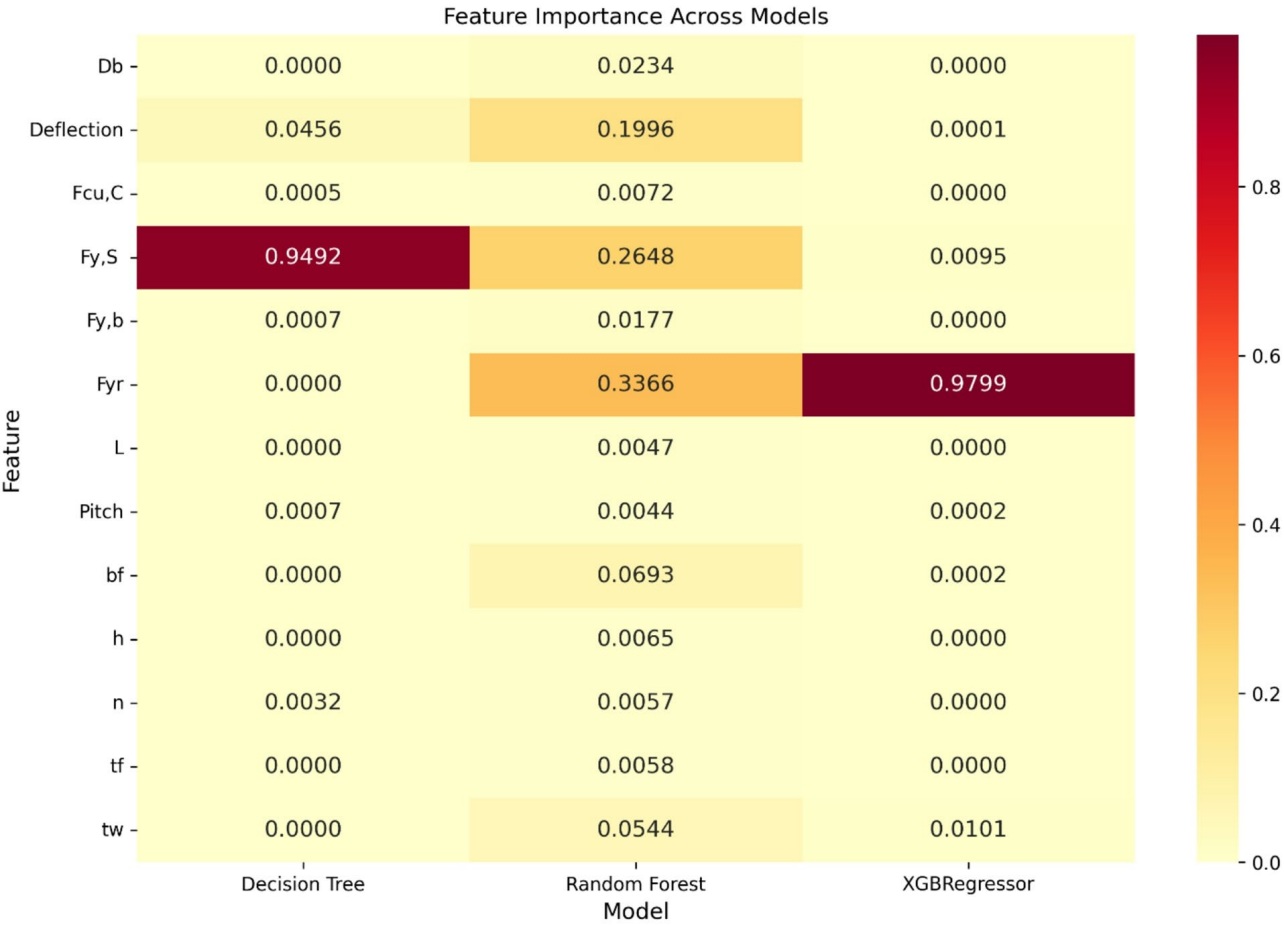
Impact of each feature on shear capacity of single stud.

Figure 8 illustrates a heatmap comparison of feature importance derived from the Decision Tree, Random Forest, and XGBoost Regressor models. The darker shades represent greater influence, while the numerical values indicate the relative contribution of each feature to the prediction of ultimate shear capacity.

The results show distinct variations across the models. In the Decision Tree, steel yield strength (F_y, S_ ​) is the most foremost feature, with a score of 0.9492, followed by deflection. For the Random Forest model, reinforcement yield strength (F_yr_ ​) was most influential (0.3366), with deflection and bolt diameter (D_b_​) also showing notable contributions. In contrast, the XGBoost Regressor highlighted reinforcement yield strength as the single most critical predictor (0.9799), far outweighing the impact of other parameters.

Geometric features such as web thickness (t_w_​) and flange width (b_f_​) demonstrated moderate importance in Random Forest but minimal influence in XGBoost. Similarly, concrete compressive strength (F_cu, C_) and span length (L) showed negligible contributions across all models.

### Interactive software development for ultimate capacity prediction

**Fig. 9:**
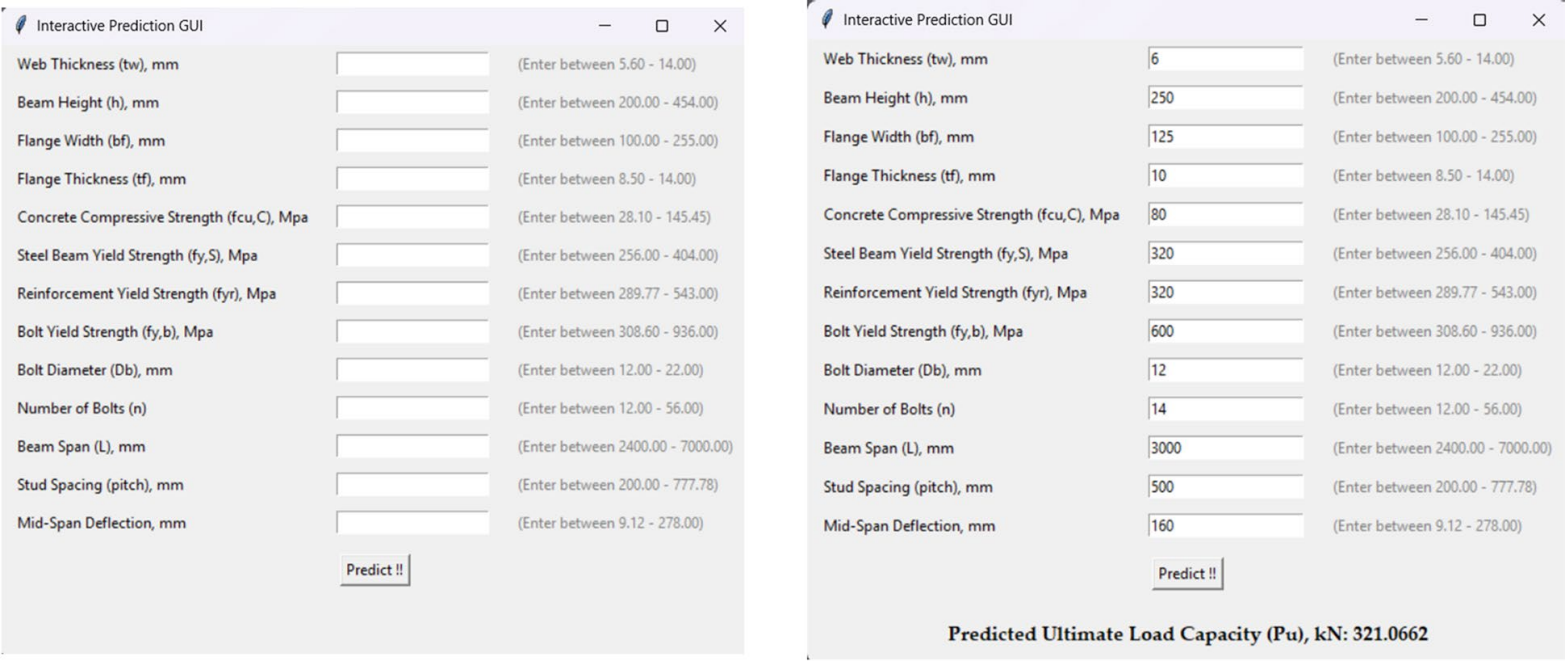
Interface of developed interactive software.

An interactive software program was created to forecast the final shear capacity of demountable shear connectors in order to turn the research’s findings into a useful tool for engineers. The program was written in Python^49^, based on the two best-performing machine learning models, Decision Tree and XGBoost, which showed excellent prediction accuracy with R^2^ values of 0.9920 and 0.9964, respectively. As shown in Fig. 9, key design variables can be entered using the software’s user-friendly and intuitive interface. The software rapidly determines the expected shear capacity after the data is submitted and shows the findings. The tool, which was created with efficiency and accessibility in mind, helps to bridge the gap between sophisticated machine learning methods and real-world engineering applications, facilitating quicker, more intelligent, and more transparent decision-making when designing demountable shear stud connectors.

## Conclusions

This work demonstrates the ability of machine learning (ML) approaches to accurately forecast the shear capacity of demountable bolted connections in steel-concrete composite beams. Eight supervised regression models were trained and tested on 146 samples to capture the complicated nonlinear interactions between connector geometry, material attributes, and structural behavior. The findings supported the efficacy of tree-based and ensemble approaches, particularly XGBoost, in producing highly accurate and interpretable predictions. The analysis also provided valuable insights into the impact of crucial design variables, which aided structural optimization and sustainable construction techniques. The following main conclusions can be drawn:

- Among eight machine learning algorithms, XGBoost achieved the highest predictive accuracy (R² = 0.996, MAE ≈ 7 kN), outperforming regularized linear and kernel-based models.
- SHAP-based interpretability highlighted bolt diameter and reinforcement yield strength as the most influential parameters governing ultimate shear capacity, consistent with experimental and numerical findings.
- The integration of advanced ML models with structural performance data offers a robust and generalizable predictive framework that improves the accuracy of connector capacity estimation.
- An interactive software tool was developed to translate trained models into practical use, enabling engineers to rapidly evaluate connector performance in real-world applications.
- Future research should focus on expanding datasets with diverse structural and environmental conditions and taking on account the effects of temperature or fatigue loads, integrating physics-informed models with machine learning, and validating the predictive framework through large-scale practical applications to enhance accuracy, generalizability, and industry adoption. Moreover, neural networks will be used to predict the behavior of shear connectors by three main parameters (Initial stiffness, strength, and ductility). Also, more work will be conducted to propose an equation for benchmarking ML predictions against some established design codes.

## Supplementary Information

Below is the link to the electronic supplementary material.

Supplementary Material 1

## Data Availability

All data generated or analyzed during this study are included in this published article as supplementary information files. In addition, any additional required data is available by the corresponding author.
